# Dissociative Identity Disorder: a case of three Selfs

**DOI:** 10.1192/j.eurpsy.2023.2027

**Published:** 2023-07-19

**Authors:** J. Bravo, I. Canelas da Silva, F. Buta

**Affiliations:** 1 Psychiatry, Hospital Vila Franca de Xira, Vila Franca de Xira

## Abstract

**Introduction:**

The DSM-5 defines dissociation as “disruption of and/or discontinuity in the normal integration of consciousness, memory, identity, emotion, perception, body representation, motor control, and behavior”. The disorders in this group include depersonalization/derealization disorder, dissociative amnesia, and dissociative identity disorder, the last being a controversial entity.

Dissociative disorders are associated with elevated levels of disability, impaired quality of life, high economic cost, and a significantly increased risk of suicide attempts.

**Objectives:**

In this work we present the case of a 21-year-old man that was assisted in the Emergency Room with dissociative symptoms. We intend to do a non-systematic review on the subject of dissociation symptoms, the psychiatric disorders in which they are present, identified risk factors, how to access the psychopathology features and the recommended treatment to best address them.

**Methods:**

For a comprehensive approach of this subject we proceeded to a non-systematic review in PubMed using the following keywords “dissociation”, “dissociative identity disorder” and “dissociative disorders”.

**Results:**

In this work we present the case of a 21-year-old man assisted in the Emergency Room describing dissociative symptoms that were suggestive of Dissociative identity disorder. He referred out-of-body experiences and a sense that he was not controlling his actions while self-injuring himself and being aggressive towards his family. He described “three Selfs”: the “Normal Self”, the “Suicidal Self” and the “Bad Self”.

Symptoms of dissociation are present in a variety of mental disorders namely depression, anxiety disorders, posttraumatic stress disorder, borderline personality disorder and eating disorders.

Dissociative disorders appear to be linked to trauma, interpersonal stress, and strongly associated with a history of chronic child abuse. An association with alexithymia, depression and suicidality were also found. Some studies found structural and functional abnormalities, particularly a reduction in grey matter volume in limbic system structures, a dysregulation of prefrontal–limbic circuitry and dysfunction of the hypothalamic–pituitary-adrenal axis.

Psychotherapy appears to be the cornerstone of treatment for dissociative disorders, namely Cognitive-Behavior therapy and Eye-movement desensitization and reprocessing.

**Conclusions:**

Symptoms of dissociation are not only present in dissociative disorders, but they may be present in almost all mental disorders. The evaluation of possible dissociative symptoms should be a part of every psychopathological assessment. There is a need for further studies to better understand this diagnostic entity and improve the therapeutic intervention.

**Disclosure of Interest:**

None Declared

